# A Genome-Wide Association Study Identifies Novel Alleles Associated with Hair Color and Skin Pigmentation

**DOI:** 10.1371/journal.pgen.1000074

**Published:** 2008-05-16

**Authors:** Jiali Han, Peter Kraft, Hongmei Nan, Qun Guo, Constance Chen, Abrar Qureshi, Susan E. Hankinson, Frank B. Hu, David L. Duffy, Zhen Zhen Zhao, Nicholas G. Martin, Grant W. Montgomery, Nicholas K. Hayward, Gilles Thomas, Robert N. Hoover, Stephen Chanock, David J. Hunter

**Affiliations:** 1 Channing Laboratory, Department of Medicine, Brigham and Women's Hospital, and Harvard Medical School, Boston, Massachusetts, United States of America; 2 Program of Molecular and Genetic Epidemiology, Harvard School of Public Health, Boston, Massachusetts, United States of America; 3 Department of Epidemiology, Harvard School of Public Health, Boston, Massachusetts, United States of America; 4 Division of Cancer Epidemiology and Genetics, National Cancer Institute, National Institutes of Health, Department of Health and Human Services, Bethesda, Maryland, United States of America; 5 BioInformed, Gaithersburg, Maryland, United States of America; 6 Department of Nutrition, Harvard School of Public Health, Boston, Massachusetts, United States of America; 7 Queensland Institute of Medical Research, Brisbane, Australia; 8 Broad Institute of Harvard and MIT, Cambridge, Massachusetts, United States of America; University of Michigan, United States of America

## Abstract

We conducted a multi-stage genome-wide association study of natural hair color in more than 10,000 men and women of European ancestry from the United States and Australia. An initial analysis of 528,173 single nucleotide polymorphisms (SNPs) genotyped on 2,287 women identified *IRF4* and *SLC24A4* as loci highly associated with hair color, along with three other regions encompassing known pigmentation genes. We confirmed these associations in 7,028 individuals from three additional studies. Across these four studies, *SLC24A4* rs12896399 and *IRF4* rs12203592 showed strong associations with hair color, with *p* = 6.0×10^−62^ and *p* = 7.46×10^−127^, respectively. The *IRF4* SNP was also associated with skin color (*p* = 6.2×10^−14^), eye color (*p* = 6.1×10^−13^), and skin tanning response to sunlight (*p* = 3.9×10^−89^). A multivariable analysis pooling data from the initial GWAS and an additional 1,440 individuals suggested that the association between rs12203592 and hair color was independent of rs1540771, a SNP between the *IRF4* and *EXOC2* genes previously found to be associated with hair color. After adjustment for rs12203592, the association between rs1540771 and hair color was not significant (*p* = 0.52). One variant in the *MATP* gene was associated with hair color. A variant in the *HERC2* gene upstream of the *OCA2* gene showed the strongest and independent association with hair color compared with other SNPs in this region, including three previously reported SNPs. The signals detected in a region around the *MC1R* gene were explained by *MC1R* red hair color alleles. Our results suggest that the *IRF4* and *SLC24A4* loci are associated with human hair color and skin pigmentation.

## Author Summary

It has been a longstanding hypothesis that human pigmentation is tightly regulated by genetic variation. However, very few genes have been identified that contain common genetic variants associated with human pigmentation. We scanned the genome for genetic variants associated with natural hair color and other pigmentary characteristics in a multi-stage study of more than 10,000 men and women of European ancestry from the United States and Australia. We identified *IRF4* and *SLC24A4* as loci highly associated with hair color, along with three other regions encompassing known pigmentation genes. Further work is needed to identify the causal variants at these loci. Improved understanding of the genetic determinants of human pigmentation may help identify the molecular mechanisms of pigmentation-associated conditions such as the tanning response and skin cancers.

## Introduction

There is substantial variation in human pigmentation within and across populations. Ultraviolet radiation (UV) exposure is the most important environmental factor influencing evolutionary selection pressure on pigmentation. In addition to UV-induced DNA damage, UVA can break down folic acid, and the major source of circulating vitamin D is synthesized in UVB-exposed skin. Because both nutrients are essential for human reproduction, it has been proposed that human pigmentation is selected, at least in part, to optimize levels of these two UV-related nutrients [1]. UV light is also the major environmental risk factor for skin cancer in humans. Red and blonde hair color, light skin pigmentation, and blue eye color are major host susceptibility factors for skin cancer [2].

Human pigmentation is a polygenic quantitative trait with high heritability [3]–[5]. A handful of genes underlying rare, extreme pigmentation phenotypes have been discovered [6], although until recently, only six genes were known to contain common genetic variants associated with human pigmentation in the normal range (*MC1R, TYR, OCA2, SLC24A5, MATP,* and *ASIP*). The proteins that these genes encode contribute to the control of melanin production and the maturation of melanosomes in melanogenesis, which determines human pigmentation. With new technologies that enable genotyping of hundreds of thousands of single nucleotide polymorphisms (SNPs), together with new insights into the structure of variation in the human genome [7], it is now possible to scan the genome in an agnostic manner in search of common genetic variants associated with human pigmentation. To identify common genetic variants associated with variation in natural diversity of human pigmentation, we performed a genome-wide association study (GWAS) of natural hair color in 2,287 U.S. women of European ancestry using data on 528,173 SNPs genotyped as part of the Cancer Genetic Markers of Susceptibility breast cancer GWAS [8]. Promising SNPs were examined in four additional studies with data on hair color and other pigmentation phenotypes: 870 U.S. women controls free of diagnosed skin cancer from a skin-cancer case-control study; 3,750 U.S. women from a diabetes case-control study; 2,405 U.S. men from a diabetes case-control study; and 1,440 parents of twins from an ongoing Australian family-based study of genetic and environmental factors contributing to the development of pigmented nevi [9].

## Results

The frequencies of pigmentary phenotypes collected in the 5 component studies are presented in Table 1. The samples were broadly similar.

**Table 1:** The distributions of human pigmentary phenotypes in the five component studies

		Initial GWAS women	skin cancer study (controls) women	NHS diabetes women	HPFS diabetes men	Australian study both genders
**hair color**		**n** (%)	**n** (%)	**n** (%)	**n** (%)	**n** (%)
	**black**	57 (2.6)	27 (3.3)	160 (4.3)	256 (10.7)	134 (9.3)
	**dark brown**	911 (41.7)	358 (43.0)	1581 (42.5)	1046 (43.7)	474 (32.9)
	**light brown**	849 (38.8)	333 (40.0)	1379 (37.1)	755 (31.5)	520 (36.1)
	**blonde**	277 (12.7)	89 (10.7)	451 (12.1)	274 (11.4)	238 (16.5)
	**red**	92 (4.2)	25 (3.0)	151 (4.1)	64 (2.7)	73 (5.1)
**skin color**						
	**fair**	-	348 (49.0)	-	-	812 (56.4)
	**medium**	-	320 (45.1)	-	-	493 (34.2)
	**olive or black**	-	42 (5.9)	-	-	135 (9.4)
**tanning ability**						
	**Practically none**	188 (8.7)	68 (8.3)	375 (10.2)	-	-
	**light tan**	454 (21.0)	173 (21.1)	850 (23.1)	621 (24.9)	-
	**average tan**	1011 (46.7)	387 (47.3)	1664 (45.1)	1089 (43.7)	-
	**deep tan**	513 (23.7)	191 (23.3)	797 (21.6)	784 (31.4)	-
**eye color**						
	**Brown/dark**	-	-	-	776 (32.3)	328 (22.8)
	**Hazel/green/medium**	-	-	-	781 (32.5)	529 (36.8)
	**Blue/light**	-	-	-	844 (35.2)	582 (40.4)

The percentages may not sum to 100 due to rounding.

**Figure 1:**
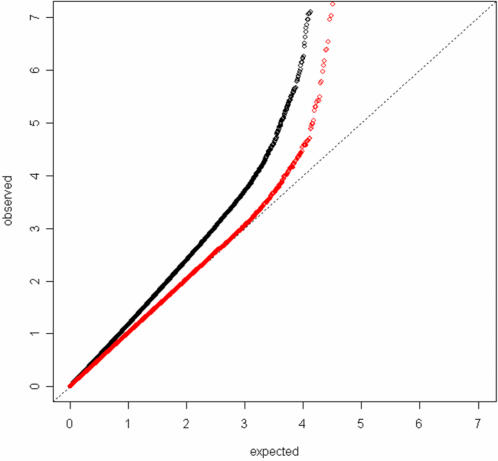
Quantile-quantile plot of the -log_10_ *p*-values from an analysis of the initial GWAS that did not adjust for principal components of genetic variation (black dots) and an analysis that did adjust for the four largest principal components (red dots). *p*-values smaller than 10^−8^ are not plotted.

We compared the distribution of observed p-values from each of the 528,173 SNPs in the GWAS with those expected under the global null hypothesis that none of the tested SNPs is associated with natural hair color (Figure 1). The distribution of the observed p-values for the crude analyses that restricted analysis to women of self-reported European ancestry but did not further adjust for potential population stratification shows evidence for systematic bias: the genomic control inflation factor for the crude analyses (the ratio of the median observed test statistic to the theoretical median) is λ_GC_ = 1.24. This systematic bias is most likely due to confounding by latent population stratification. Hair color varies along a light-dark gradient from northern to southern Europe, so it will be associated with any SNP marker whose minor allele frequency also varies along a North-South gradient, even if that marker is not in linkage disequilibrium (LD) with a causal hair-color locus [10]. Adjusting for the top four principal components of genetic variance [11] eliminated most of the apparent residual confounding due to population stratification (λ_GC_ = 1.02 for the adjusted analyses); further control for up to 50 principal components did not alter the λ_GC_. All of the association results from the initial GWAS reported below are from analyses that adjusted for the top four principal components of genetic variation.

**Figure 2:**
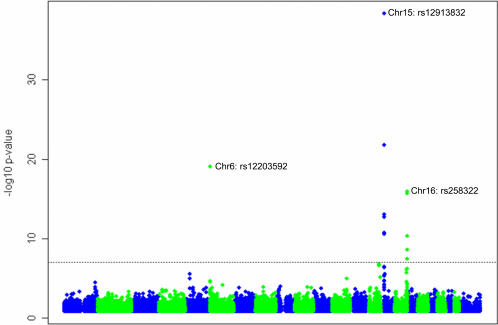
-log10 *p*-values from the primary test of association with hair color in the initial GWAS, by position along chromosome. Only *p*-values smaller than 0.05 are plotted.

The GWAS identified several genomic locations as potentially associated with hair color (Figure 2). Of 528,173 SNPs tested, the 38 SNPs with the most extreme p-values associated with hair color are listed in Table 2.

**Table 2:** Thirty-eight SNPs with the smallest p-values of the 528,173 tested for association of hair color in the initial GWAS of in 2,287 women of European ancestry

						hair color (black to red)				non-red hair color (black to blonde)			red vs. non-red color		
SNP	Chromosome	Location	GeneNeighborhood	WT/VT	MAF	beta	s.e.	p value	Selected for replication	beta	s.e.	p value	beta	s.e.	p value
rs12913832	15	26039213	HERC2	G/A	0.25	−0.37	0.03	4.3E-39	Y	−0.38	0.02	2.7E-56	−0.20	0.18	0.25
rs1667394	15	26203777	HERC2	T/C	0.17	−0.33	0.03	1.5E-22	LD with rs8039195	−0.33	0.03	1.2E-29	−0.30	0.22	0.18
rs12203592	6	341321	IRF4	C/T	0.17	−0.31	0.03	8.2E-20	Y	−0.36	0.03	6.6E-36	0.42	0.18	0.02
rs258322	16	88283404	MC1R	C/T	0.09	0.36	0.04	8.0E-18	Y	0.07	0.04	0.08	1.89	0.18	5.0E-27
rs4785763	16	88594437	MC1R	C/A	0.33	0.23	0.03	1.7E-17	Y	0.07	0.02	4.8E-03	1.75	0.17	5.7E-24
rs6497268	15	26012308	OCA2	C/A	0.18	−0.25	0.03	8.5E-15	Y	−0.24	0.03	9.5E-18	−0.35	0.22	0.10
rs8039195	15	26189679	HERC2	C/T	0.15	−0.28	0.04	1.9E-14	Y	−0.33	0.03	7.4E-30	−0.24	0.23	0.30
rs11855019	15	26009415	OCA2	A/G	0.14	−0.26	0.04	2.1E-12	Y	−0.26	0.03	4.4E-16	−0.33	0.24	0.18
rs11636232	15	26060221	HERC2	C/T	0.40	0.18	0.03	3.2E-12	Y	0.16	0.02	2.1E-12	0.38	0.15	0.01
rs8049897	16	88551703	MC1R	G/A	0.15	0.24	0.04	4.8E-12	Y	0.08	0.03	0.01	1.36	0.16	1.4E-16
rs4238833	16	88578190	MC1R	T/G	0.37	0.18	0.03	5.6E-12	Y	0.04	0.02	0.09	1.62	0.17	1.7E-20
rs4408545	16	88571529	MC1R	T/C	0.50	0.16	0.03	3.0E-10	Y	0.04	0.02	0.06	1.54	0.19	1.9E-15
rs7204478	16	88322986	MC1R	C/T	0.44	0.15	0.03	1.0E-08	Y	0.02	0.02	0.31	1.52	0.18	4.7E-17
rs4904866	14	91838256		C/T	0.43	0.14	0.03	3.0E-08	LD with rs12896399	0.17	0.02	1.2E-13	−0.10	0.15	0.53
rs12896399	14	91843416	SLC24A4	T/G	0.43	0.14	0.03	3.0E-08	Y	0.16	0.02	1.7E-13	−0.10	0.15	0.51
rs7174027	15	26002360	OCA2	G/A	0.11	−0.22	0.04	5.0E-08	Y	−0.22	0.04	2.6E-10	−0.31	0.27	0.25
rs7183877	15	26039328	HERC2	C/A	0.08	−0.26	0.05	6.0E-08	Y	−0.24	0.04	3.5E-09	−0.27	0.32	0.39
rs7196459	16	88668978	MC1R	G/T	0.08	0.25	0.05	1.0E-07	Y	0.05	0.04	0.26	1.40	0.19	3.1E-13
rs164741	16	88219799	MC1R	C/T	0.30	0.14	0.03	1.2E-07	Y	0.01	0.02	0.62	1.30	0.16	4.6E-17
rs7188458	16	88253985	MC1R	G/A	0.43	0.13	0.03	3.1E-07	Y	0.01	0.02	0.74	1.49	0.18	5.8E-17
rs8033165	15	26805134		C/T	0.46	0.12	0.02	4.3E-07	Y	0.10	0.02	1.9E-06	0.36	0.14	0.01
rs35391	5	33991430	MATP	C/T	0.03	−0.41	0.08	4.6E-07	LD with rs28777	−0.43	0.07	7.9E-10	−0.17	0.51	0.74
rs7495174	15	26017833	OCA2	A/G	0.08	−0.24	0.05	7.1E-07	Y	−0.25	0.04	2.6E-09	−0.24	0.31	0.44
rs1635168	15	26208861	HERC2	C/A	0.08	−0.25	0.05	9.0E-07	LD with rs8028689	−0.27	0.04	6.6E-10	−0.18	0.32	0.57
rs8007923	14	103726412	KIF26A	T/C	0.47	−0.12	0.03	1.2E-06	Y	−0.10	0.02	6.0E-06	−0.28	0.15	0.07
rs10861741	12	106353647		C/T	0.15	0.17	0.04	1.8E-06	Y	0.12	0.03	1.6E-04	0.55	0.19	3.1E-03
rs28777	5	33994716	MATP	A/C	0.03	−0.36	0.08	1.9E-06	Y	−0.37	0.06	1.1E-08	−0.31	0.51	0.54
rs9806558	15	23458783		C/T	0.33	−0.13	0.03	3.4E-06	Y	−0.09	0.02	1.1E-04	−0.40	0.17	0.02
rs9392056	6	463078	EXOC2	A/G	0.38	0.12	0.03	4.1E-06	LD with rs6918152	0.10	0.02	4.5E-06	0.19	0.15	0.22
rs4778211	15	25872900	OCA2	C/A	0.16	−0.16	0.03	4.1E-06	Y	−0.15	0.03	3.5E-07	−0.17	0.22	0.44
rs2493040	6	480839	EXOC2	G/A	0.32	0.13	0.03	4.3E-06	LD with rs6918153	0.11	0.02	3.3E-06	0.16	0.16	0.30
rs6918152	6	487159	EXOC2	G/A	0.37	0.12	0.03	5.1E-06	Y	0.11	0.02	2.5E-06	0.14	0.15	0.34
rs2353033	16	87913062	MC1R	T/C	0.43	0.12	0.03	5.2E-06	Y	0.04	0.02	0.09	0.92	0.16	6.1E-09
rs12142165	1	224689937	OBSCN	T/C	0.35	−0.12	0.03	6.3E-06	Y	−0.09	0.02	1.2E-04	−0.41	0.17	0.02
rs7195066	16	88363824	MC1R	C/T	0.31	−0.12	0.03	9.5E-06	Y	−0.01	0.02	0.64	−1.83	0.29	3.9E-10
rs2241039	16	88615938	MC1R	C/T	0.38	−0.11	0.03	1.1E-05	Y	−0.05	0.02	0.04	−0.90	0.19	1.1E-06
rs8028689	15	26162483	HERC2	T/C	0.06	−0.24	0.05	1.1E-05	Y	−0.25	0.05	1.5E-07	−0.24	0.35	0.49
rs16950987	15	26199823	HERC2	G/A	0.06	−0.24	0.05	1.2E-05	LD with rs8028689	−0.24	0.05	1.7E-07	−0.24	0.35	0.49

The p-values are based on primary association test (including women with red hair) adjusted for top four principal components of genetic variance.

The regression parameter beta refers to the mean change in pigmentation scoring (or change in log odds of red hair for red hair analyses) per copy of the SNP minor allele.

We selected 31 of these 38 SNPs for further study in an independent sample. The remaining seven SNPs were in strong LD (r^2^>0.8) with one of these 31 SNPs (Table 2). The sample consisted of 870 controls of European ancestry from a nested case-control study of skin cancer within the Nurses' Health Study (NHS). Thirty of the 31 attempted SNPs were genotyped successfully.

Twenty-two of these 30 SNPs showed very strong evidence for association with natural hair color (p<9.5×10^−8^ = 0.05/528,173) in a pooled analysis of the initial GWAS and the validation sample (Table 3). Of the remaining eight SNPs, three showed very strong evidence for association with hair color either after excluding women with red hair or when comparing women with red hair to those without (Table 3). The associations between these 30 SNPs and with tanning ability and skin color are presented in Table S1 and Table S2.

**Table 3:** Thirty-one SNPs among the controls in the skin cancer study within the NHS, and pooled with the GWAS data

						hair color (black to red)						non-red hair color blonde) (black to										
SNP	Chromosome	Position	GeneNeighborhood	WT/VT	in skin cancer set beta	s.e.	p value	in pooled set beta	s.e.	p value	in skin cancer set beta	s.e.	p value	in pooled set beta	s.e.	p value	in skin cancer set beta	s.e.	p value	in pooled set beta	s.e.	p value
rs12142165	1	224689937	OBSCN	T/C	−0.02	0.04	0.64	−0.10	0.02	4.1E-05	−0.01	0.04	0.84	−0.07	0.02	9.8E-04	−0.19	0.33	0.56	−0.37	0.15	0.01
rs28777	5	33994716	MATP	A/C	−0.39	0.11	2.6E-04	−0.46	0.06	8.9E-14	−0.41	0.09	2.0E-05	−0.46	0.05	1.2E-17	0.12	0.72	0.86	−0.18	0.41	0.66
rs12203592	6	341321	IRF4	C/T	−0.38	0.05	7.1E-13	−0.31	0.03	8.5E-28	−0.40	0.05	7.6E-18	−0.36	0.02	7.1E-49	0.12	0.37	0.75	0.36	0.16	0.02
rs6918152	6	487159	EXOC2	G/A	0.09	0.04	0.04	0.11	0.02	5.3E-07	0.09	0.04	0.02	0.11	0.02	6.1E-08	−0.02	0.31	0.94	0.11	0.14	0.42
rs10861741	12	106353647		C/T	−0.02	0.06	0.71	0.12	0.03	1.3E-04	−0.03	0.05	0.56	0.08	0.03	5.3E-03	0.12	0.41	0.77	0.47	0.17	5.4E-03
rs12896399	14	91843416	SLC24A4	T/G	0.15	0.04	2.7E-04	0.17	0.02	3.0E-14	0.15	0.04	4.0E-05	0.18	0.02	8.3E-21	0.06	0.29	0.83	−0.07	0.14	0.63
rs8007923	14	103726412	KIF26A	T/C	0.05	0.04	0.25	−0.07	0.02	1.5E-03	0.03	0.04	0.43	−0.06	0.02	2.7E-03	0.28	0.29	0.34	−0.16	0.13	0.23
rs9806558	15	23458783		C/T	−0.01	0.04	0.78	−0.08	0.02	4.7E-04	−0.02	0.04	0.62	−0.06	0.02	3.5E-03	0.09	0.29	0.75	−0.28	0.15	0.06
rs4778211	15	25872900	OCA2	C/A	−0.11	0.06	0.05	−0.16	0.03	2.2E-07	−0.10	0.05	0.07	−0.15	0.03	3.7E-08	−0.31	0.44	0.48	−0.20	0.20	0.32
rs7174027	15	26002360	OCA2	G/A	−0.09	0.06	0.14	−0.25	0.03	2.7E-13	−0.13	0.06	0.02	−0.25	0.03	9.1E-17	0.42	0.40	0.30	−0.12	0.22	0.59
rs11855019	15	26009415	OCA2	A/G	−0.14	0.06	0.02	−0.29	0.03	6.1E-20	−0.16	0.05	2.5E-03	−0.28	0.03	1.5E-24	0.21	0.40	0.60	−0.20	0.21	0.33
rs6497268	15	26012308	OCA2	C/A		failed the assay																
rs7495174	15	26017833	OCA2	A/G	−0.16	0.07	0.03	−0.30	0.04	9.9E-14	−0.20	0.06	2.1E-03	−0.30	0.03	4.0E-18	0.46	0.45	0.31	−0.06	0.25	0.82
rs12913832	15	26039213	HERC2	G/A	−0.44	0.05	1.1E-22	−0.44	0.02	9.9E-78	−0.43	0.04	6.3E-28	−0.44	0.02	4.2E-103	−0.27	0.37	0.46	−0.22	0.16	0.18
rs7183877	15	26039328	HERC2	C/A	−0.32	0.08	3.0E-05	−0.29	0.04	2.0E-12	−0.32	0.07	3.1E-06	−0.28	0.04	5.1E-15	−0.18	0.60	0.76	−0.25	0.28	0.37
rs11636232	15	26060221	HERC2	C/T	0.19	0.04	1.0E-05	0.23	0.02	1.7E-25	0.15	0.04	1.4E-04	0.20	0.02	1.1E-24	0.63	0.29	0.03	0.43	0.13	1.2E-03
rs8028689	15	26162483	HERC2	T/C	−0.18	0.08	0.02	−0.31	0.04	4.0E-12	−0.25	0.07	5.0E-04	−0.32	0.04	9.2E-17	0.61	0.46	0.18	0.02	0.27	0.95
rs8039195 (rs916977) *	15	26189679	HERC2	C/T	−0.35	0.05	6.3E-11	−0.36	0.03	1.6E-32	−0.36	0.05	1.9E-14	−0.35	0.03	6.3E-42	0.06	0.38	0.89	−0.16	0.20	0.40
rs8033165	15	26805134		C/T	0.11	0.04	9.0E-03	0.15	0.02	2.0E-12	0.08	0.04	0.02	0.12	0.02	5.4E-11	0.37	0.29	0.21	0.36	0.13	5.2E-03
rs2353033	16	87913062	MC1R	T/C	0.13	0.04	1.2E-03	0.14	0.02	8.0E-10	0.08	0.04	0.02	0.06	0.02	1.7E-03	0.74	0.29	0.01	0.88	0.14	2.8E-10
rs164741	16	88219799	MC1R	C/T	0.22	0.05	1.4E-06	0.17	0.02	2.1E-13	0.11	0.04	0.01	0.04	0.02	0.04	1.57	0.33	1.4E-06	1.36	0.14	5.2E-22
rs7188458	16	88253985	MC1R	G/A	0.16	0.04	2.7E-04	0.16	0.02	4.0E-12	0.07	0.04	0.06	0.04	0.02	0.05	1.27	0.33	1.4E-04	1.44	0.16	3.8E-20
rs258322	16	88283404	MC1R	C/T	0.30	0.07	1.0E-05	0.36	0.04	2.2E-23	0.18	0.06	3.6E-03	0.12	0.04	6.6E-04	1.21	0.34	4.4E-04	1.75	0.15	7.2E-30
rs7204478	16	88322986	MC1R	C/T	0.22	0.04	3.5E-08	0.17	0.02	7.5E-15	0.11	0.04	2.6E-03	0.05	0.02	0.02	1.99	0.40	6.1E-07	1.61	0.16	1.3E-22
rs7195066	16	88363824	MC1R	C/T	−0.12	0.05	6.5E-03	−0.11	0.02	2.5E-06	−0.03	0.04	0.52	−0.01	0.02	0.74	−3.11	1.01	2.2E-03	−2.00	0.28	8.5E-13
rs8049897	16	88551703	MC1R	G/A	0.10	0.06	0.11	0.22	0.03	3.0E-12	0.00	0.05	0.98	0.06	0.03	0.02	1.11	0.36	2.1E-03	1.31	0.15	1.3E-18
rs4408545	16	88571529	MC1R	T/C	0.08	0.04	0.04	0.15	0.02	1.0E-11	−0.02	0.04	0.69	0.03	0.02	0.08	1.73	0.40	1.5E-05	1.57	0.17	1.4E-19
rs4238833	16	88578190	MC1R	T/G	0.12	0.05	7.7E-03	0.17	0.02	3.9E-13	0.02	0.04	0.68	0.03	0.02	0.15	1.53	0.35	1.0E-05	1.60	0.16	8.2E-25
rs4785763	16	88594437	MC1R	C/A	0.13	0.04	2.7E-03	0.21	0.02	1.7E-19	0.00	0.04	0.92	0.05	0.02	0.01	1.89	0.34	4.3E-08	1.78	0.16	1.5E-30
rs2241039	16	88615938	MC1R	C/T	0.00	0.04	0.97	−0.08	0.02	5.7E-04	0.04	0.04	0.28	−0.01	0.02	0.47	−0.60	0.33	0.07	−0.84	0.16	2.4E-07
rs7196459	16	88668978	MC1R	G/T	0.32	0.07	2.0E-05	0.28	0.04	5.0E-12	0.15	0.07	0.02	0.09	0.04	0.02	1.47	0.34	1.3E-05	1.42	0.17	2.0E-17

*: Chromosome

**: The SNP rs8039195 failed the assay and the SNP rs916977 was genotyped instead (R square = 0.85).

The regression parameter beta refers to the mean change in pigmentation scoring (or change in log odds of red hair for red hair analyses) per copy of the SNP minor allele.

**Figure 3:**
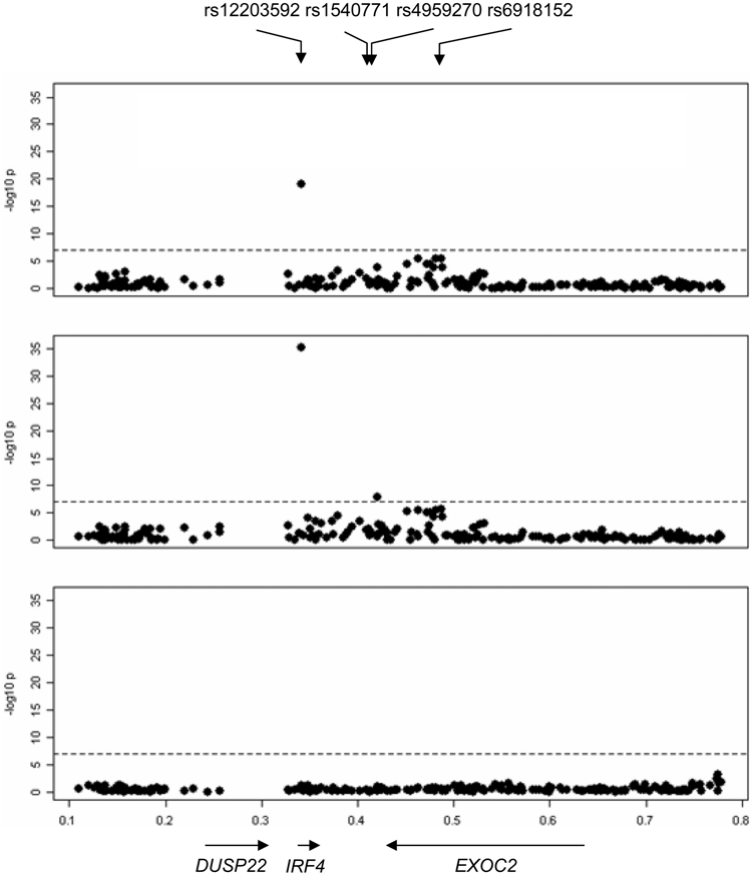
Association analysis of SNPs across IRF4 region. -log10 *p*-values from the primary test of association with hair color in the initial GWAS (top panel), from the test of association with hair color excluding individuals with red hair (middle), and from the test of association with red hair versus non-red hair (bottom). The region plotted spans Chr6:95272.789899 (NCBI build 35).

The SNP rs12203592 in intron 4 of the *IRF4* gene was strongly associated with hair color in the initial GWAS and validation study (black to red, pooled p value for trend = 8.5×10^−28^; black to blonde, pooled p value for trend = 7.1×10^−49^). The percentage of residual variation in hair color from black to blonde explained by this SNP after controlling for the top four principal components of genetic variation was 7.0%. This SNP is within 69.7 kb of two SNPs (rs4959270 and rs1540771) that were identified by a recent GWAS of natural hair color in women of European ancestry resident in Iceland [12]. However, neither of these variants, which lie between *EXOC2* (*SEC5L1*) and *IRF4,* was as strongly associated with natural hair color in our initial GWAS as the *IRF4* SNP rs12203592 (Figure 3). In our GWAS, the p values for association between hair color (black to blonde) and rs4959270 and rs1540771 were 2.9×10^−4^ and 0.007, respectively, and those for tanning ability were 0.002 and 0.001, respectively. In fact, the p-value for association between rs12203592 and natural hair color was more than 13 orders of magnitude smaller than the p-value for any other SNP on chromosome 6. This should not be taken as evidence that the loci that influence hair color in Iceland are different from those for the rest of Europe; rather, the previous GWAS may have failed to identify rs12203592 because this SNP is not on the Illumina HumanHap300 array used in that study, while it is on the Illumina HumanHap550 array used here.

We genotyped rs12203592 in an additional 6,155 individuals of predominantly European ancestry from the United States, including 3,750 women from the NHS and 2,405 men from the Health Professionals Follow-up Study (HPFS), and in an additional 1440 individuals of European ancestry from Australia (Queensland Institute of Medical Research (QIMR)). The association with hair color showed strong reproducibility; the independent p-values for association with hair color (black to blonde) in these three follow-up studies was 3.2×10^−40^, 2.8×10^−35^, and 4.6×10^−23^ respectively, and the pooled p-value across all five studies was 1.5×10^−137^ (Table 4). There was no statistical evidence for heterogeneity in the magnitude or direction of the hair color-minor allele correlation across the studies. The rs12203592 SNP was also highly associated with skin color (6.2×10^−14^), eye color (6.1×10^−13^), and tanning ability (3.9×10^−89^) in subsets of these individuals for which this information was available (Table 4). This variant allele was associated with lighter skin color, less tanning ability, and blue/light eye color (Table 5).

**Table 4:** Association of IRF4 SNP rs12203592 and SLC24A4 SNP rs12896399 with pigmentary phenotypes in five studies

	hair color (black to blonde)			skin color			eye color			tanning ability		
	beta	s.e.	p value	beta	s.e.	p value	beta	s.e.	p value	beta	s.e.	p value
***IRF4*** ** SNP rs12203592**												
**Initial GWAS**	−0.36	0.03	6.6E-36	-	-	-	-	-	-	0.34	0.03	2.5E-23
**skin cancer study**	−0.40	0.05	7.6E-18	0.17	0.04	8.0E-05	-	-	-	0.40	0.06	1.7E-12
**NHS diabetes**	−0.32	0.02	3.2E-40	-	-	-	-	-	-	0.38	0.03	5.1E-44
**HPFS diabetes**	−0.39	0.03	2.8E-35	-	-	-	0.18	0.03	5.3E-09	0.22	0.03	1.6E-14
**Australian study**	−0.38	0.04	4.6E-23	0.17	0.03	1.4E-09	0.13	0.03	1.0E-04	-	-	-
**pooled data**	−0.35	0.01	1.5E-137	0.18	0.02	6.2E-14	0.17	0.02	6.1E-13	0.37	0.02	3.9E-89
**pooled data without initial GWAS**	−0.35	0.02	2.0E-106	0.18	0.02	6.2E-14	0.17	0.02	6.1E-13	0.36	0.02	2E-61
***SLC24A4*** ** SNP rs12896399**												
**Initial GWAS**	0.16	0.02	1.7E-13	-	-	-	-	-	-	0.07	0.03	0.01
**skin cancer study**	0.15	0.04	4.0E-05	0.06	0.03	0.07	-	-	-	0.03	0.04	0.45
**NHS diabetes**	0.19	0.02	7.2E-27	-	-	-	-	-	-	0.02	0.02	0.41
**HPFS diabetes**	0.22	0.03	1.2E-17	-	-	-	0.11	0.02	2.9E-06	0.01	0.02	0.73
**pooled data**	0.19	0.01	6.0E-62	-	-	-	-	-	-	0.04	0.01	0.01
**pooled data without initial GWAS**	0.20	0.01	9.3E-47	-	-	-	-	-	-	0.02	0.02	0.27

**Table 5:** Distributions of IRF4 rs12203592 and SLC24A4 rs12896399 with pigmentary phenotypes in the pooled five studies

	IRF4 RS12203592	wt (%)	het (%)	var (%)	SLC24A4 RS12896399	wt (%)	het (%)	var (%)
**hair color**								
	**black**	260 (3.8)	276 (9.6)	63 (16.5)	**black**	222 (7.8)	184 (4.4)	50 (2.9)
	**dark brown**	2485 (36.5)	1486 (51.6)	184 (48.2)	**dark brown**	1360 (47.9)	1804 (43.3)	555 (32.3)
	**light brown**	2709 (39.7)	857 (29.7)	104 (27.2)	**light brown**	923 (32.5)	1558 (37.4)	699 (40.7)
	**blonde**	1139 (16.7)	125 (4.3)	14 (3.7)	**blonde**	219 (7.7)	474 (11.4)	355 (20.7)
	**red**	224 (3.3)	137 (4.8)	17 (4.5)	**red**	116 (4.1)	149 (3.6)	57 (3.3)
**skin color**								
	**fair**	650 (49.3)	402 (58.1)	86 (86.0)	**fair**	102 (47.2)	164 (47.5)	71 (54.6)
	**medium**	533 (40.4)	252 (36.4)	12 (12.0)	**medium**	96 (44.4)	163 (47.2)	55 (42.3)
	**olive or black**	135 (10.2)	38 (5.5)	2 (2.0)	**olive or black**	18 (8.3)	18 (5.2)	4 (3.1)
**tanning ability**								
	**Practically none**	625 (10.4)	431 (18.1)	122 (40.0)	**Practically none**	382 (13.4)	563 (13.5)	237 (13.8)
	**light tan**	872 (14.6)	471 (19.8)	72 (23.6)	**light tan**	411 (14.4)	682 (16.3)	341 (19.8)
	**average tan**	2764 (46.2)	1058 (44.4)	94 (30.8)	**average tan**	1330 (46.7)	1902 (45.5)	726 (42.2)
	**deep tan**	1724 (28.8)	424 (17.8)	17 (5.6)	**deep tan**	724 (25.4)	1034 (24.7)	415 (24.1)
**eye color**								
	**Brown/dark**	761 (32.0)	265 (24.2)	14 (8.7)	**Brown/dark**	249 (33.3)	352 (34.2)	102 (24.3)
	**Hazel/green/medium**	797 (33.5)	392 (35.7)	57 (35.4)	**Hazel/green/medium**	283 (37.8)	302 (29.3)	132 (31.4)
	**Blue/light**	822 (34.5)	440 (40.1)	90 (55.9)	**Blue/light**	216 (28.9)	375 (36.4)	186 (44.3)

Note: Percentages represent distribution of pigmentation traits within each genotype.

On the same chromosome, 145.8 kb centromeric from the *IRF4* rs12203592, the SNP rs6918152 in the *EXOC2* gene was associated with hair color (black to blond) in the initial GWAS, the NHS skin cancer controls, and the Australian samples (Tables 3 and 6). These two SNPs are in very weak LD (r^2^ = 0.04). Genotypes for the SNP rs1540771 previously reported by Sulem et al. [12] were available in the initial scan and the Australian samples. In a mutually adjusted multivariable regression of rs12203592, rs6918152, rs1540771, the strength of the association between the first SNP with hair color was attenuated but remained significant (p = 2.7×10^−17^ for *IRF4* rs12203592 in the Australian samples) (Table 6). The previously reported SNP rs1540771 was not significant (p>0.05) after adjustment for the other SNPs, and association between rs6918152 and hair color was much weaker in the GWAS and no longer significant in the Australian samples after adjustment. While the *IRF4* SNP rs12203592 was also associated with skin color, eye color and tanning ability, the *EXOC2* SNP rs6918152 was not associated with these phenotypes. These results suggest that the *IRF4* SNP rs12203592 is most likely to be in strong LD with the causal variant in this region.

**Table 6:** Association between SNPs in EXOC2 and IRF4 and hair color (black to blonde)

		marginal analysis			multivariable analysis	
	beta	s.e.	p value	beta	s.e.	p value
**Initial GWAS**						
rs12203592	−0.36	0.03	6.6E-36	−0.35	0.03	8.0E-29
rs6918152	0.11	0.02	2.5E-06	0.07	0.02	3.6E-03
rs1540771	0.06	0.02	7.2E-03	−0.03	0.02	0.16
**Australian Study**						
rs12203592	−0.38	0.04	4.6E-23	−0.36	0.04	2.7E-17
rs6918152	0.14	0.03	2.0E-05	0.05	0.03	0.12
rs1540771	0.12	0.03	3.3E-04	0.02	0.04	0.57
**Pooled**						
rs12203592	−0.35	0.02	3.1E-52	−0.35	0.03	2.7E-44
rs6918152	0.12	0.02	2.7E-09	0.06	0.02	1.4E-03
rs1540771	0.09	0.02	3.4E-06	−0.01	0.02	0.52

The multivariable analysis mutually adjusted for the three SNPs.

The rs12896399 SNP 15.5 kb upstream of the *SLC24A4* gene was highly associated with light hair color, and relatively weakly associated with less tanning ability in the pooled analysis of four studies (p = 6.0×10^−62^ for hair color, and p = 0.01 for tanning ability). The percentage of residual variation in hair color from black to blonde explained by this SNP after controlling for the top four principal components of genetic variation was 2.6%. This variant was also associated with blue/light eye color (p = 2.9×10^−6^ in the HPFS set). The *SLC24A4* gene belongs to a family of potassium-dependent sodium/calcium exchangers. At least two other members of this family are associated with skin pigmentation. The *SLC24A5* gene was recently shown to be involved in skin pigmentation in both zebrafish and humans [13].

Another member of this family, *MATP (SLC45A2)*, is a pigmentation gene transcriptionally regulated by *MITF* [14],[15]. We identified the SNP rs28777 in the *MATP* gene from the GWAS, and the association with hair color was replicated in the controls of the skin cancer study (pooled P value = 8.9×10^−14^). This SNP was also associated with skin color (pooled P value = 9.5×10^−4^) and tanning ability (pooled P value = 2.2×10^−10^). Three SNPs in the *MATP* gene have been associated with human pigmentation: rs16891982 (Phe374Leu), rs26722 (Glu272Lys), and rs13289 C/G (-1721 in the promoter region) [16],[17]. We genotyped these three SNPs in the controls of the skin cancer study. None of the three previously reported SNPs were in LD with rs28777 (r^2^≤0.01), which is an intronic SNP. A multivariable analysis mutually adjusting for rs28777, rs16891982, rs26722, and rs13289 simultaneously showed that only rs16891982 remained significant in the model (P = 0.036 for hair color (black to blonde), P = 0.016 for tanning ability, and P = 0.0009 for skin color) and other SNPs became non-significant (p>0.05). These data suggested that rs16891982 is most likely to be the causal variant or in strong LD with the causal variant in the *MATP* gene.

Eleven SNPs spanning 1 Mb on chromosome 15 were strongly associated with hair color in the initial GWAS. These SNPs were located in the *OCA2* 5′ regulatory region and the *HERC2* gene region and included the 3 SNPs reported previously with eye color: rs7495174, p = 7.1×10^−7^; rs6497268, p = 8.5×10^−15^; and rs11855019, p = 2.1×10^−12^) [18]. In an analysis mutually adjusting for all 11 SNPs simultaneously, only the *HERC2* SNP rs12913832 (not on the HumanHap 300 version used in Sulem et al. [12]) remained significantly associated with hair color (p = 2.73×10^−32^) and tanning ability (p = 3.03×10^−9^). The associations between all other SNPs and hair color became non-significant (p>0.05). This suggested that the SNP rs12913832 was most likely to be in strong linkage disequilibrium with the causal variant in this region. The percentage of residual variation in hair color from black to blonde explained by this SNP after controlling for the top four principal components of genetic variation was 10.7%.

We observed 12 SNPs on chromosome 16 associated with hair color in the GWAS, spanning >756 kb. The *MC1R* gene, well established to be associated with red hair color, is located within this region. We had previously genotyped 7 common *MC1R* variants among the NHS skin cancer controls [19]. The analysis mutually adjusting for all 19 SNPs in the controls of the skin cancer study indicates that the signals that we detected in this region were mainly due to the three *MC1R* red hair color alleles (Arg151Cys, Arg160Trp, and Asp294His) (Table S3). The pairwise LD among these 19 SNPs was very low (the pattern of LD across these 19 SNPs is shown in Figure S1).

## Discussion

It has been a longstanding hypothesis that human pigmentation is tightly regulated by genetic variation. However, very few genes have been identified that contain common genetic variants associated with human pigmentation. We conducted a genome-wide association study of hair color and identified several new variants associated with variation in hair color, skin color, eye color, and tanning ability among individuals of European ancestry.

Among the loci identified from our GWAS, the *IRF4* and *SLC24A4* loci had not been linked to human pigmentation before we began our study. Recently, Sulem et al. [12] reported a pigmentation GWAS using 316,515 SNPs in the Icelandic population. The associations of hair color in our GWAS with the 60 SNPs reported by Sulem et al. are listed in Table S4. These authors identified two SNPs (rs4959270 and rs1540771) between the *EXOC2* and *IRF4* genes in relation to freckles, hair color, and skin sensitivity to sun [12]. We identified a SNP in intron 4 of the *IRF4* gene, not genotyped by Sulem et al. [12], with a much stronger association than they observed with hair color and tanning ability. The *IRF4* gene product is a member of the interferon regulatory factor family of transcription factors [20]–[23], which are involved in the regulation of gene expression in response to interferon and other cytokines. The *IRF4* gene encodes a B-cell proliferation/differentiation protein, which has been proposed as a sensitive and specific marker for conventional primary and metastatic melanomas and benign melanocytic nevi [24].

The SNP rs12896399 upstream of the *SLC24A4* gene showed strong association with hair color in our study and that of Sulem et al. [12]. In addition, we identified three chromosomal regions adjacent to the previously known pigmentation genes: *MC1R, OCA2*, and *MATP*. The *MC1R* gene encodes a 317-amino acid 7-pass-transmembrane G protein coupled receptor and has been shown as the rate-limiting step in the activation of the cAMP pathway in terms of melanin production. Although the LD between the MC1R variants and surrounding highly significant SNPs was relatively low, the multivariable models mutually adjusting for all surrounding SNPs suggests that the signals that we identified on chromosome 16 were explained by the functional variants in the *MC1R* gene.

A previous report showed that three SNPs in intron 1 of the *OCA2* gene were associated with eye, skin, and hair color [18]. We identified a SNP (rs12913832) in the upstream *HERC2* gene with a much stronger association with hair color and tanning ability. Because the *HERC2* gene has not been linked to human pigmentation to our knowledge, the SNP may be involved in the regulation of the expression or the function of the *OCA2* gene. Similarly, Sulem et al. identified rs1667394 (∼165 kb upstream from rs12913832, r^2^ = 0.58) as the strongest hit in this region in their study [12]. However, we found a stronger association of rs12913832 with hair color than that of rs1667394 in our study.

Similar to our findings, Sulem et al. reported associations in the regions encompassing *MC1R*, *OCA2*, and *SLC24A4*. In addition, they reported loci in two established pigmentation genes, *TYR* and *KITLG*, which were not among the 38 SNPs with the strongest associations with hair color (black to red) that we sought to genotype in additional samples. The p-value for the association between *KITLG* rs12821256 and hair color (black to red) in the initial GWAS was 0.0002; the p-value for the association with hair color excluding women with red hair (black to blonde) was 1.28×10^−8^. The p-values for association between *TYR* rs1393350 and hair color coded as black to red or black to blonde were 0.05 and 0.02, respectively. We additionally identified another previously reported pigmentation gene from our GWAS, the *MATP* gene that was not reported by Sulem et al. [12]. In our analysis of testing the trend across hair color from black to blonde, there were no other loci reaching genome-wide significance level.

Two of the four regions we found to be associated with variation in hair color among Europeans without red hair (*MATP* and *HERC2*/*OCA2*) show strong evidence of recent positive selection, based on a comparison of allele frequencies across samples from three continental populations (Africa, Asia, and Europe) [25]. Both of the markers we identified in the two remaining regions showed significant differences in allele frequency across the HapMap CEU, CHB, JPT and YRI panels: the *IRF4* SNP rs12203592 was monomorphic in CHB, JPT and YRI panels (the minor allele in Europeans was absent from these samples); the minor allele among Europeans for *SLC24A4* SNP rs12896399 (G) was the major allele for the CHB and YRI panels, with the G allele frequency in the YRI sample being above 99%. Moreover, all of the markers with strongest association with hair color in these four regions were significantly associated with one or more of the top four principal components of genetic variation (Table S5), suggesting that allele frequencies for these markers also vary among European populations. Because we adjusted for latent population structure using these four principal components—and there are multiple lines of evidence suggesting these regions influence hair color among Europeans—we believe it unlikely that the strong associations we see between these markers and hair color are solely due to population stratification bias. Rather it is likely that differences in the distribution of hair color across Europe are due in part to differences in allele frequencies at these loci and other as-yet-unknown loci. Taken together, these four regions explain approximately 21.9% of the residual variation in hair color (black-blond) after adjusting for the top four principal components of genetic variation. (Conversely, after adjusting for these four regions, the top four principal components of genetic variation explain 2.6% of the residual variation in hair color.)

In our study of men and women of European ancestry we focused on the most statistically significant associations from our GWAS among women, identifying the *IRF4* variant as reproducibly associated with human pigmentation. Further work is needed to identify the causal variant at this locus. Because a subset of true associations would be weakly associated with outcome in any given GWAS, large-scale replication is necessary for confirmation, and some true associations may be missed if they are not carried forward into replication studies. In this regard, the precomputed rankings and *P* values for all the SNPs included in the GWAS conducted in the NHS are freely available (http://www.channing.harvard.edu/nhs/publications/index.shtml) for others to use in subsequent studies.

## Materials and Methods

### Nurses' Health Study (NHS)

The NHS was established in 1976, when 121,700 female U.S. registered nurses between the ages of 30 and 55, residing in 11 larger U.S. states, completed and returned the initial self-administered questionnaire on their medical histories and baseline health-related exposures, forming the basis for the NHS cohort. Biennial questionnaires with collection of exposure information on risk factors and (every 4 years since 1980) nutritional assessments have been collected prospectively. Along with exposures every 2 years, outcome data with appropriate follow-up of reported disease events, including melanoma and non-melanoma skin cancers, are collected. Overall, follow-up has been very high; after more than 20 years approximately 90% of participants continue to complete questionnaires. From May 1989 through September 1990, we collected blood samples from 32,826 participants in the NHS cohort. Subsequent follow-up has been greater than 99% for this subcohort. The information on natural hair color at age of 20 and childhood and adolescence tanning ability were collected in the 1982 questionnaire.

### Initial GWAS

We initially performed genotyping in a nested case-control study of postmenopausal invasive breast cancer within the Nurses' Health Study (NHS) cohort [26] using the Illumina HumanHap550 array, as part of the National Cancer Institute's Cancer Genetic Markers of Susceptibility (CGEMS) Project [8]. We performed our initial genome-wide analysis on 528,173 SNPs in 2,287 women [8]. All cases and controls were self-described as being of European ancestry. Four samples were excluded because of evidence of intercontinental admixture. Controlling for breast cancer case-control status made no material difference to the GWAS results. Information on natural hair color at age 20 was collected in the NHS main questionnaire and grouped into five categories (black, dark brown, light brown, blonde, and red).

Detailed methods related to the initial GWAS were published previously [8], including genotyping and quality control, initial assessment of sample completion rates, assessment of SNP call rates, concordance rate, deviation from Hardy–Weinberg proportions in control DNA, and final sample selection and exclusion for association analysis.

### The Controls in the Skin Cancer Nested Case-Control Study within the NHS

The promising SNPs from the initial GWAS were further genotyped among 870 controls in the skin cancer nested case-control study within the NHS. The distribution of risk factors for skin cancer in the subcohort of those who donated blood samples was very similar to that in the overall cohort [2]. A common control series was randomly selected from participants who gave a blood sample and were free of diagnosed skin cancer up to and including the questionnaire cycle in which the corresponding case was diagnosed.

### Health Professionals Followup Study (HPFS)

In 1986, 51,529 men from all 50 U.S. States in health professions (dentists, pharmacists, optometrists, osteopath physicians, podiatrists, and veterinarians) aged 40–75 answered a detailed mailed questionnaire, forming the basis of the study. Between 1993 and 1994, 18,159 study participants provided blood samples by overnight courier. The information on natural hair color and eye color was collected in the 1988 questionnaire, and the information on tanning ability was asked in the 1992 questionnaire.

### The Diabetes Nested Case-Control Studies within the NHS and HPFS

Two additional studies were used to genotype novel pigmentation loci: 3,750 samples from the nested case-control study of diabetes in the NHS and 2,405 samples from the nested case-control study of diabetes in the HPFS [27]. All samples that we used were cases and controls from these two studies. Cases were incident cases of diabetes after blood collection, and controls were matched on age. Controlling for case-control status made no material difference to the results.

There was no sample overlap among the initial GWAS, the skin cancer case-control study, and the two diabetes case-control studies. The study protocol was approved by the Institutional Review Board of Brigham and Women's Hospital and Harvard School of Public Health. Informed consent was obtained from all patients.

### Australian Study from the Queensland Institute of Medical Research (QIMR)

The Australian sample comprised 1,442 parents of twins taking part in a long-running study of melanoma risk factors [9],[18],[28],[29]. Participants rated their own hair color (at age 20 years) on a five-point classification (blonde, light brown, dark brown, black, red), eye color (blue/grey, green/hazel, brown), and skin color (light, medium, or dark).

### Statistical Analysis

For the primary analysis of hair color we regressed an ordinal coding for hair color (1 = black; 2 = dark brown; 3 = light brown; 4 = blonde; and 5 = red) on an ordinal coding for genotype (0, 1 or 2 copies of SNP minor allele) separately for each SNP that passed quality control filters [8]. Crude analyses that did not adjust for any other variables showed evidence of systematic bias (see results); as this bias was greatly reduced by adjusting for the four largest principal components of genetic variation, all subsequent association analyses in the initial GWAS included these four components in the regression model. These principal components were calculated for all individuals on the basis of ca. 10,000 unlinked markers using the EIGENSTRAT software [8],[11]. The top four eigenvectors were chosen on the basis of significant (p<0.05) Tracy-Wisdom tests [30]. Adjusting for up to the top 50 principal components did not further reduce the genomic control inflation factor λ_GC_. We chose markers for genotyping in subsequent validation studies based on the p-values for association from the primary analysis. Partial correlation coefficients (i.e., adjusted r^2^ or the percent of residual variance explained by the SNP marker) were calculated from the linear regressions adjusted for the top four principal components of genetic variation.

There is some evidence that determinants of hair color may act along two phenotypic axes: red hair color versus non-red color and light-dark variation among those without red hair. For example, alleles at the *MC1R* locus primarily determine presence or absence of red hair [31]. Hence, we conducted further analyses among individuals without red hair and comparing those with red hair to those without to evaluate whether discovered loci act on one or both phenotypic axes. We regressed the ordinal coding for hair color on minor allele counts excluding the individuals with red hair and used logistic regression to test the association between the ordinal genotype coding and a binary red-hair phenotype (red vs. non-red hair color). The regression parameter beta refers to the mean change in hair color scoring (or change in log odds of red hair for red hair analyses) per copy of the SNP minor allele.

We also used linear regression to test association between minor allele counts and self-reported tanning in response to sunlight (1 = deep tan, 2 = average tan, 3 = light tan, 4 = no tan), eye color (1 = brown/dark, 2 = hazel/green/medium, and 3 = blue/light), and skin color (1 = black, 2 = medium, and 3 = fair). Pooled analyses of multiple studies were conducted by merging data sets and including separate baseline parameters for each study.

### Genotyping in Followup Studies

The TaqMan/BioTrove assays on the 31 SNPs in the skin cancer controls were performed at the Dana Farber/Harvard Cancer Center Polymorphism Detection Core (primers and probe sequences are available on request). Two loci (*IRF4* rs12203592 and *SLC24A4* rs12896399) were further genotyped in diabetes samples in the NHS and HPFS studies using the Taqman assay. Laboratory personnel were blinded to the case-control status, and 10% blinded quality control samples were inserted to validate genotyping procedures; concordance for the blinded samples was 100%. Primers, probes, and conditions for genotyping assays are available upon request.

For the Australian study, genotyping was performed as a single multiplex reaction on the Sequenom high-throughput genotyping platform on *IRF4* SNP rs12203592 and *EXOC2* rs6918152 and the best pigmentation-associated SNPs in the region of 6p25 (rs1540771) reported by Sulem et al. [12].

## Supporting Information

Figure S1 The LD pattern of SNPs around the MC1R locus on Chromosome 16.
(0.06 MB PDF)

Table S1 Associations between the 30 most significant SNPs, which were identified in the GWAS of hair color, and tanning ability in the GWAS, the skin cancer controls, and the pooled data.
(0.02 MB XLS)

Table S2 Associations between the 30 most significant SNPs, which were identified in the GWAS of hair color, and skin color in the skin cancer controls.
(0.02 MB XLS)

Table S3 Associations between SNPs around the MC1R locus on chromosome 16 and hair color and tanning ability.
(0.02 MB XLS)

Table S4 The associations between hair color and the 60 SNPs in Supplementary Table 1 of Sulem et al. [12].
(0.03 MB XLS)

Table S5 The associations between SNPs in the MATP, IRF4, SLC24A4, and HERC2 genes and EVs (from 1 to 10).
(0.02 MB XLS)

## References

[1] Jablonski NG, Chaplin G (2000). The evolution of human skin coloration.. J Hum Evol.

[2] Han J, Colditz GA, Hunter DJ (2006). Risk factors for skin cancers: a nested case-control study within the Nurses' Health Study.. Int J Epidemiol.

[3] Clark P, Stark AE, Walsh RJ, Jardine R, Martin NG (1981). A twin study of skin reflectance.. Ann Hum Biol.

[4] Frisancho AR, Wainwright R, Way A (1981). Heritability and components of phenotypic expression in skin reflectance of Mestizos from the Peruvian lowlands.. Am J Phys Anthropol.

[5] Harrison GA, Owen JJ (1964). Studies on the inheritance of human skin colour.. Ann Hum Genet.

[6] Sturm RA (2006). A golden age of human pigmentation genetics.. Trends Genet.

[7] Frazer KA, Ballinger DG, Cox DR, Hinds DA, Stuve LL (2007). A second generation human haplotype map of over 3.1 million SNPs.. Nature.

[8] Hunter DJ, Kraft P, Jacobs KB, Cox DG, Yeager M (2007). A genome-wide association study identifies alleles in FGFR2 associated with risk of sporadic postmenopausal breast cancer.. Nat Genet.

[9] Duffy DL, Box NF, Chen W, Palmer JS, Montgomery GW (2004). Interactive effects of MC1R and OCA2 on melanoma risk phenotypes.. Hum Mol Genet.

[10] Campbell CD, Ogburn EL, Lunetta KL, Lyon HN, Freedman ML (2005). Demonstrating stratification in a European American population.. Nat Genet.

[11] Price AL, Patterson NJ, Plenge RM, Weinblatt ME, Shadick NA (2006). Principal components analysis corrects for stratification in genome-wide association studies.. Nat Genet.

[12] Sulem P, Gudbjartsson DF, Stacey SN, Helgason A, Rafnar T (2007). Genetic determinants of hair, eye and skin pigmentation in Europeans.. Nat Genet.

[13] Lamason RL, Mohideen MA, Mest JR, Wong AC, Norton HL (2005). SLC24A5, a putative cation exchanger, affects pigmentation in zebrafish and humans.. Science.

[14] Newton JM, Cohen-Barak O, Hagiwara N, Gardner JM, Davisson MT (2001). Mutations in the human orthologue of the mouse underwhite gene (uw) underlie a new form of oculocutaneous albinism, OCA4.. Am J Hum Genet.

[15] Du J, Fisher DE (2002). Identification of Aim-1 as the underwhite mouse mutant and its transcriptional regulation by MITF.. J Biol Chem.

[16] Graf J, Hodgson R, van Daal A (2005). Single nucleotide polymorphisms in the MATP gene are associated with normal human pigmentation variation.. Hum Mutat.

[17] Graf J, Voisey J, Hughes I, van Daal A (2007). Promoter polymorphisms in the MATP (SLC45A2) gene are associated with normal human skin color variation.. Hum Mutat.

[18] Duffy DL, Montgomery GW, Chen W, Zhao ZZ, Le L (2007). A three-single-nucleotide polymorphism haplotype in intron 1 of OCA2 explains most human eye-color variation.. Am J Hum Genet.

[19] Han J, Kraft P, Colditz GA, Wong J, Hunter DJ (2006). Melanocortin 1 receptor variants and skin cancer risk.. Int J Cancer.

[20] Grossman A, Mittrucker HW, Nicholl J, Suzuki A, Chung S (1996). Cloning of human lymphocyte-specific interferon regulatory factor (hLSIRF/hIRF4) and mapping of the gene to 6p23-p25.. Genomics.

[21] Eisenbeis CF, Singh H, Storb U (1995). Pip, a novel IRF family member, is a lymphoid-specific, PU.1-dependent transcriptional activator.. Genes Dev.

[22] Matsuyama T, Grossman A, Mittrucker HW, Siderovski DP, Kiefer F (1995). Molecular cloning of LSIRF, a lymphoid-specific member of the interferon regulatory factor family that binds the interferon-stimulated response element (ISRE).. Nucleic Acids Res.

[23] Yamagata T, Nishida J, Tanaka S, Sakai R, Mitani K (1996). A novel interferon regulatory factor family transcription factor, ICSAT/Pip/LSIRF, that negatively regulates the activity of interferon-regulated genes.. Mol Cell Biol.

[24] Sundram U, Harvell JD, Rouse RV, Natkunam Y (2003). Expression of the B-cell proliferation marker MUM1 by melanocytic lesions and comparison with S100, gp100 (HMB45), and MelanA.. Mod Pathol.

[25] Barreiro LB, Laval G, Quach H, Patin E, Quintana-Murci L (2008). Natural selection has driven population differentiation in modern humans.. Nat Genet.

[26] Tworoger SS, Eliassen AH, Sluss P, Hankinson SE (2007). A prospective study of plasma prolactin concentrations and risk of premenopausal and postmenopausal breast cancer.. J Clin Oncol.

[27] Qi L, van Dam RM, Meigs JB, Manson JE, Hunter D (2006). Genetic variation in IL6 gene and type 2 diabetes: tagging-SNP haplotype analysis in large-scale case-control study and meta-analysis.. Hum Mol Genet.

[28] Palmer JS, Duffy DL, Box NF, Aitken JF, O'Gorman LE (2000). Melanocortin-1 receptor polymorphisms and risk of melanoma: is the association explained solely by pigmentation phenotype?. Am J Hum Genet.

[29] Zhu G, Montgomery GW, James MR, Trent JM, Hayward NK (2007). A genome-wide scan for naevus count: linkage to CDKN2A and to other chromosome regions.. Eur J Hum Genet.

[30] Patterson N, Price AL, Reich D (2006). Population structure and eigenanalysis.. PLoS Genet.

[31] Rees JL (2004). The genetics of sun sensitivity in humans.. Am J Hum Genet.

