# Supplementary figures and images for: A Genome-Wide Association Study Identifies Novel Alleles Associated with Hair Color and Skin Pigmentation
Source: PLoS Genet. 2008 May 16;4(5):e1000074. doi: 10.1371/journal.pgen.1000074 (PMC2367449; doi:10.1371/journal.pgen.1000074)

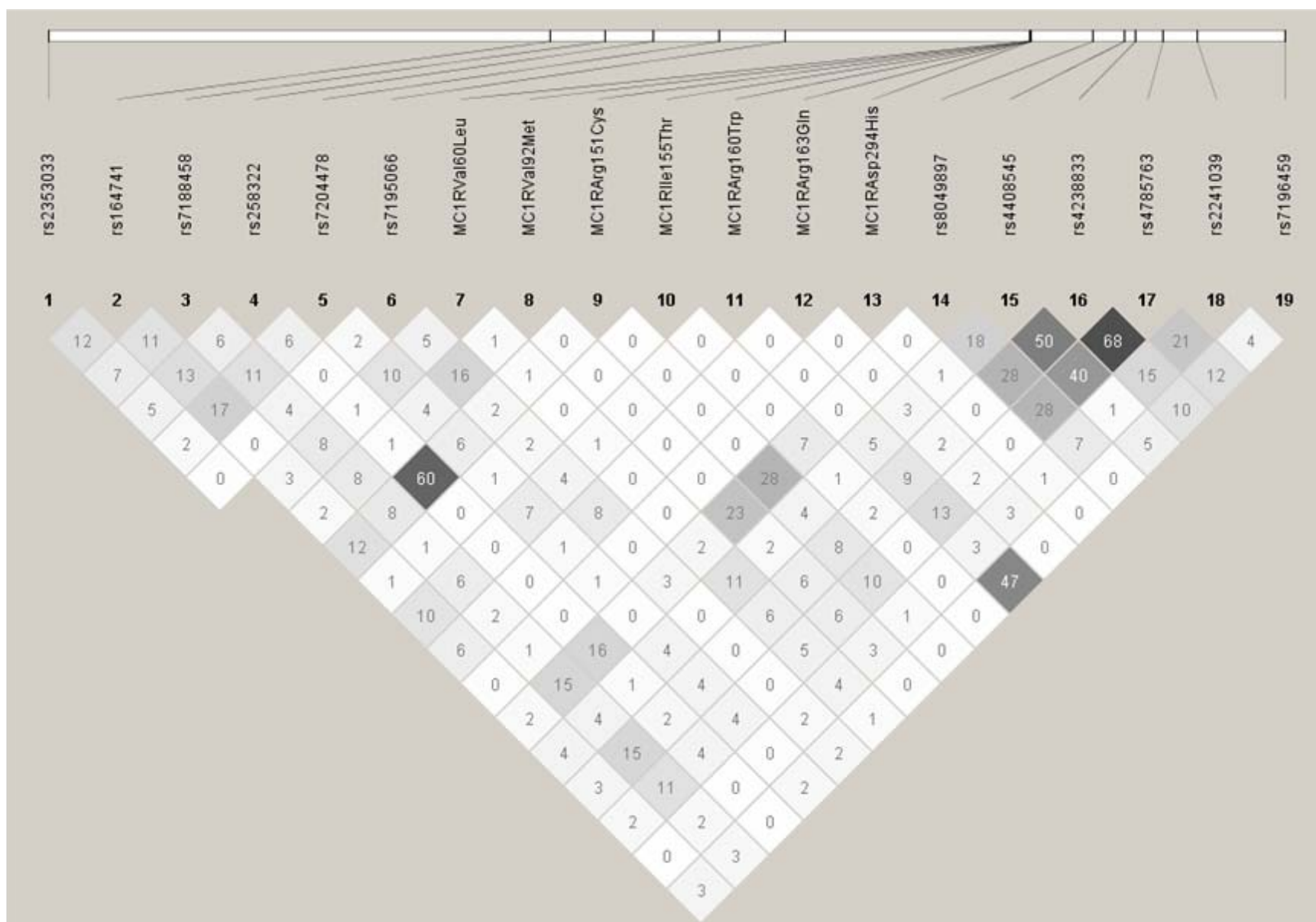

Supplement: Figure S1 — The LD pattern of SNPs around the MC1R locus on Chromosome 16. (0.06 MB PDF) [file pgen.1000074.s001.pdf]
